# mSphere of Influence: 3-D Culture Models Influence Studies on Epstein-Barr Virus Molecular Pathogenesis in the Epithelium

**DOI:** 10.1128/mSphere.00954-20

**Published:** 2020-09-23

**Authors:** K. H. Y. Shair

**Affiliations:** a Cancer Virology Program, University of Pittsburgh Medical Center, University of Pittsburgh, Pittsburgh, Pennsylvania, USA; b Department of Microbiology and Molecular Genetics, University of Pittsburgh, Pittsburgh, Pennsylvania, USA

**Keywords:** 3-D culture, Epstein-Barr virus, air-liquid interface culture models, nasopharyngeal carcinoma

## Abstract

Kathy Shair works in the field of Epstein-Barr virus (EBV)-associated cancers, with emphasis on nasopharyngeal carcinoma (NPC). In this mSphere of Influence article, she reflects on how the paper “Efficient replication of Epstein-Barr virus in stratified epithelium *in vitro*” by Temple et al. (R. M. Temple, J. Zhu, L. Budgeon, N. D. Christensen, et al., Proc Natl Acad Sci U S A 111:16544–16549, 2014, https://doi.org/10.1073/pnas.1400818111) has influenced her work on EBV molecular pathogenesis in the nasopharynx by highlighting the importance of using three-dimensional (3-D) culture models to study epithelial infection.

## COMMENTARY

Are virus-associated cancers linked to a defect in virus replication? The unlicensed expression of viral oncogenes driven by integration into the host genome is one manner by which virus replication can be disabled but cause havoc to the host cell by promoting cancer. What is enigmatic about Epstein-Barr virus (EBV)-associated cancers is that the EBV genome is not integrated, existing as multicopy episomes that favor latency instead of lytic infection. Many EBV studies related to epithelial infection are performed in two-dimensional (2-D) monolayer culture. Adherent monolayer cultures reproduce EBV latency but cannot recapitulate lytic reactivation in response to cellular differentiation—the physiological stimulus for EBV reactivation in epithelial cells (1–3). Reactivation, and the failure to reactivate, is therefore fundamental to our understanding of EBV molecular pathogenesis in preneoplastic mechanisms. In the article “Efficient replication of Epstein-Barr virus in stratified epithelium *in vitro*” from the laboratory of C. Sample, her group uncovers what happens to *de novo* EBV infection upon differentiation of primary oral keratinocytes modeled in organotypic raft culture (2). For many years, I have studied the oncogenic properties of the EBV oncoprotein, latent membrane protein 1 (LMP1), without a true appreciation for its significance in the context of EBV infection. This ultimately fueled my interest to pursue better 3-D culture models to extrapolate the role of LMP1 in epithelial culture models (4).

Organotypic rafts are a form of 3-D cell culture that can be established with primary or immortalized keratinocytes to model the stratified squamous epithelium. To trigger cellular differentiation, organotypic rafts are lifted to the air-liquid interface (ALI) on metal grids or on commercial transwell membranes. There are other forms of 3-D culture, such as suspension in hydrogels or nonadherent culture dishes, but only ALI culture methods can generate self-organizing organoids that simulate the diversified cell types of the epithelium. Three-dimensional culture models are sophisticated, and establishing conditions for *de novo* infection of primary keratinocytes is arguably the most intricate but likely the most authentic. The *de novo* infection conditions in primary organotypic rafts mirror the shuttling of EBV from B-cells to epithelial cells, reflecting EBV’s alternate cell tropism (5). What makes the paper by Temple et al. (2) remarkable is that *de novo* EBV infection of primary keratinocytes produces a highly productive infection to levels not typically observed with recombinantly infected EBV-positive cell lines. To carry out the infection, EBV B-cell inoculum is applied to the scored (or wounded) surface of an organotypic raft that is undergoing differentiation. Several days later (4 to 6 days postinfection), the organotypic raft completes differentiation. Wounding of the raft is not necessary but encourages virus adsorption and provides access to the basal layer where latently infected cells (should they exist) are expected to reside (6). To convincingly demonstrate that the reactivation is not abortive and that infectious virus is produced, Temple et al. (2) run the gamut of EBV molecular virology assays, showing expression of lytic genes localizing to the suprabasal (differentiated) layers, EBV genome replication, and production of infectious EBV. Together, these results strengthen the claim that EBV favors reactivation in differentiating epithelia.

What was striking to me was that there was no evidence of latently infected cells, which would most likely occur in the basal layer. Thus, it appears that failure to reactivate in the stratified epithelium is an uncommon event. Given that EBV-infected epithelial cells are difficult to find *in vivo* (7) and since EBV infection of epithelial cells (primary or immortalized) is inefficient in 2-D culture, the studies by Temple et al. (2) shed light on EBV molecular pathogenesis in ways that could otherwise not be revealed by conventional methods.

Several laboratories have since used variations of organotypic rafts with immortalized keratinocytes to elucidate the molecular pathogenesis of EBV in stratified epithelium (3, 8–10). Altogether, these studies have influenced the EBV field by providing a method in which to elucidate the regulatory (cellular and viral) factors that contribute to EBV reactivation in response to a differentiation stimulus. I had hypothesized that EBV reactivation models could also be applied to nasopharyngeal cells. This is provided that the conditions for culturing, differentiating, and infecting primary nasopharyngeal cells could be established. I first embarked on this idea by establishing conditions to study EBV reactivation in a nasopharyngeal carcinoma (NPC)-derived cell line, HK1 (11). Somewhat confounding was that authenticated NPC-derived cell lines are rare and that native EBV infection is often lost upon serial culture, unless maintained as a xenograft (12, 13). At the time that I initiated these studies, HK1 cells were the only authenticated NPC-derived cell line devoid of HeLa contaminants that could continually carry EBV by recombinant infection and selection (14). Using such HK1-EBV cells induced to reactivate by ALI culture, we discovered by knockdown that LMP1 is required for early stages of EBV reactivation, before expression of immediate-early genes (15, 16). This function of LMP1 in lytic reactivation also occurs in oral keratinocytes (17), giving us the confidence that we could rely on conclusions drawn from different ALI systems. In the past year, two additional EBV-infected NPC-derived cell lines (C17 and NPC43) have been established (13, 18), and it might be possible to extrapolate ALI-induced reactivation studies to these additional cell lines, as well as nasopharynx-derived organotypic rafts.

What has spurred new interest is to ask whether we could study EBV *de novo* infection in primary nasopharyngeal cells by ALI culture. Primary cells offer the unique perspective of host genetic variability, which would allow evaluation of possible variation in EBV susceptibility as well as the type of infection outcome (lytic or latent). There is a longstanding debate as to whether EBV and/or host genetics contributes to NPC risk, and epidemiology data would support that both are possible (19, 20). The holy grail would be to develop an experimental system where one could manipulate the EBV genome to test EBV genetic variants on cells from different donors. For the past few years, my lab has worked on adapting the ALI culture method to study EBV *de novo* infection in primary pseudostratified airway epithelial cells derived from the nasopharynx, using conditionally reprogrammed cells (CRCs) (21). One interesting finding was that we found variability in EBV susceptibility between different donors, even when the cells were exposed to the same inoculum in the same experiment. Furthermore, latently infected cells were observed in some donor cultures, but lytic infection could occur in cultures from another donor. Because the primary CRCs could be banked and revived as low-passage-number cells, we were confident that such donor variation in susceptibility and pathogenesis was reproducible. Much remains to be determined using such complementary 3-D culture systems, but I believe that we are presented with a new era because it is now possible to study EBV molecular pathogenesis in all the cell types (stratified and pseudostratified) of the nasopharynx.
